# Event-Related Potentials in ADHD Associated With Tuberous Sclerosis Complex: A Possible Biomarker of Symptoms Severity?

**DOI:** 10.3389/fneur.2020.00546

**Published:** 2020-07-10

**Authors:** Romina Moavero, Sara Marciano, Stefano Pro, Donata De Stefano, Federico Vigevano, Paolo Curatolo, Massimiliano Valeriani

**Affiliations:** ^1^Child Neurology and Psychiatry Unit, Systems Medicine Department, Tor Vergata University of Rome, Rome, Italy; ^2^Child Neurology Unit, Neuroscience and Neurorehabilitation Department, Bambino Gesù Children's Hospital, IRCCS, Rome, Italy; ^3^Center for Sensory Motor Interaction Aalborg University, Aalborg, Denmark

**Keywords:** tuberous sclerosis complex, ADHD, ERP, MMN, P300, attention

## Abstract

**Background and Aim:** Tuberous sclerosis complex (TSC) is associated with a high rate of attention deficit-hyperactivity disorder (ADHD), usually with more severe symptoms than in idiopathic cases. Event-related potentials have been used in idiopathic ADHD, and they have been proposed as a possible biomarker of symptoms severity. Aim of this study was to investigate event-related potential (ERP) characteristics in patients with ADHD secondary to TSC, compared to patients with drug-naive idiopathic ADHD and healthy controls (HCs), to investigate whether (1) distinct clinical features can be due to different pathophysiological mechanisms, and (2) ERPs may reliably predict ADHD symptoms severity in TSC.

**Materials and Methods:** We enrolled 13 patients with idiopathic ADHD (iADHD), 6 patients with ADHD associated with TSC (tscADHD), and 14 age-matched HCs (7–17 years). All of them underwent ERP recording, with mismatch negativity (MMN) preceding the P300 recording. All patients underwent neurocognitive evaluations.

**Results:** Mismatch negativity latency was shorter in iADHD (*P* = 0.04) and tscADHD (*P* = 0.06) than in HC, with no difference between patients' groups. Mismatch negativity amplitude was significantly higher in patients (both iADHD and tscADHD) than in HC. The P300 amplitude was significantly lower in iADHD patients than in both tscADHD patients (*P* = 0.03) and HCs (*P* < 0.001). No difference was found between tscADHD patients and HCs (*P* = 0.2).

**Conclusion:** While patients with iADHD present lower P300 amplitude than HC, in tscADHD patients P300 amplitude was not different from that in HC, suggesting that in TSC P300 amplitude does not really reflect symptom severity.

## Introduction

Tuberous sclerosis complex (TSC) is an autosomal dominant multisystem disease characterized by hamartomas in several organs and systems (1). Central nervous system involvement represents the main source of morbidity for patients affected by this complex disease and includes epilepsy and a variety of neuropsychiatric disorders (2). Attention-deficit hyperactivity disorder (ADHD) is a neuropsychiatric condition highly prevalent in the general population (3%−7%) (3, 4), but with a significantly higher ratio in patients with TSC, affecting ~30–50% of patients (2). Attention-deficit hyperactivity disorder is characterized by inattention, hyperactivity, and impulsive behavior (5)Core symptoms of ADHD include specific deficits in executive functions, including inhibition, and in attentional processes such as vigilance, sustained, divided, and selective attention (6). Attention-deficit hyperactivity disorder occurs in up to 50% of patients with TSC, being 10 times more prevalent in TSC than in the general population (7). Despite this frequent association, the real pathophysiological mechanisms underlying this comorbidity are not completely understood, but cortical tubers, frontal epileptiform abnormalities, and the genetic mutation *per se* are believed to play a role (2, 8). In TSC, ADHD often co-occurs with other neuropsychiatric disorders, including intellectual disability and autism spectrum disorder (7). Clinical experience suggests that symptoms of ADHD in children with TSC are usually more severe and tend to present lower benefits to pharmacological and non-pharmacological treatments when compared to idiopathic ADHD.

Attention and inhibition, which are core aspects in ADHD, can be studied with neurophysiological techniques, including event-related potentials (ERPs), which in the last decades have been widely used in this population, revealing abnormalities in the attention-dependent processing (9). The P300 component is an endogenous positive potential peaking 300 ms after a stimulus and generated by several cortical and subcortical structures (10, 11). It is believed to reflect executive and attentional function, including the updating of working memory, event categorization, and attentional resource allocation, as well as attentional reorientation (12). Moreover, P300 has been proposed to reflect late-stage monitoring of outcomes related to inhibitory processes (13). Therefore, its amplitude seems to be related with the allocation of attentional resources, whereas the latency reflects the stimulus evaluation time (14). Mismatch negativity (MMN) is an ERP component occurring ~100–200 ms after the onset of a deviant stimulus, therefore before P300 (15), and it represents an automatic cerebral discrimination process, not under attentive control (16).

Different reports suggest that children with ADHD present lower amplitude and higher latency P300 (17), so that some authors proposed to use this ERP component as a marker of disease severity (18). The available data concerning MMN are more conflicting with different studies failing to find statistically significant differences between children with ADHD and healthy subjects (19). Event-related potential components can be modified by ADHD pharmacotherapies, such as atomoxetine and methylphenidate, which increase the P300 (20) and MMN (21) amplitudes.

The aim of this study was to investigate ERP characteristics in patients with ADHD secondary to TSC, compared to patients with drug-naive idiopathic ADHD and healthy controls (HCs), to investigate whether distinct clinical features can be due to different pathophysiological mechanisms.

## Materials and Methods

### Participants

We enrolled patients with idiopathic ADHD (iADHD) and with ADHD associated with TSC (tscADHD), and age-matched HCs. Inclusion criteria for patients with ADHD (with or without TSC) were diagnosis of ADHD according to the *Diagnostic and Statistical Manual of Mental Disorders, Fifth Edition* (DSM-5) criteria and age 7–17 years. Moreover, iADHD patients did not have to present any other neurological or medical condition associated with ADHD. Exclusion criteria were cognitive impairment (intelligence quotient <70), psychiatric comorbidity, and sensory deficits that could interfere with behavioral performances or with electrophysiological results. If ADHD was pharmacologically treated, children were asked to withdraw medications for 48 h before the neurophysiological and neuropsychological examination. Healthy controls were children and adolescents without a history of neurological or neuropsychiatric conditions. All patients and healthy subjects had had a normal audiological evaluation before the study. All participants' caregivers signed an informed consent form. The study was approved by the local ethical board.

### ERP Recording and Analysis

For ERP recording, subjects were comfortably seated in a quiet room. Mismatch negativity recording preceded the P300 recording in all our children and adolescents.

Auditory stimuli were sinusoidal tones (10-ms duration, 2-ms rise and 2-ms fall time, 85-dB SPL of intensity), presented binaurally via headphones. Frequent 750-Hz tones and deviant 500-Hz tones were delivered with a probability of 85 and 15%, respectively. A fixed interstimulus interval (ISI) of 1 s and an ISI variable between 0.8 and 1.2 s were used for MMN and P300 recording, respectively.

Event-related potentials were recorded from three scalp electrodes, located at Fz, Cz, and Pz positions of the 10–20 International System. A further electrode placed in the outer cantus of the right eye recorded the electro-oculogram (EOG). Reference was at the nose. Electroencephalogram (EEG) sampling rate was of 1,024 Hz, and the analysis time was 1,000 ms, including 100 ms of prestimulus delay. The amplifier bandpass was 0.1 to 30 Hz (24-dB roll-off). An automatic artifact-rejection system excluded from the average all runs containing transients exceeding ± 150 μV at any recording channel, including the EOG. Averages of 15 trials (deviant stimuli) were used for ERP measurements.

#### MMN Recording

Mismatch negativity was recorded after 100 acoustic stimuli. Children were instructed to read a novel; thus, they did not pay attention to the acoustic stimulation. They were required to summarize the novel in a short briefing following the stimulation.

#### P300 Recording

Children underwent a block of ~100 acoustic stimuli. They were instructed to count the number of infrequent tones mentally. No motor response was required. Averages in which counting mistake had exceeded 10% would not have been considered in the data analysis.

#### ERP Analysis

The N1 and P2 latencies and the peak-to-peak N2/P2 amplitude were measured in the Cz traces recorded to deviant stimuli. For MMN labeling, difference traces, obtained by subtracting the frequent stimuli from deviant stimuli traces, were calculated. In Fz difference trace, MMN latency and amplitude were measured at the peak and from the baseline, respectively. P300 latency and amplitude were measured in the Pz trace to deviant stimuli at the peak and from the baseline, respectively.

### Cognitive and Neuropsychological Examination

All patients underwent the administration of an extensive battery of tests for the assessment of cognitive functioning and neuropsychological phenotype. This battery included the following tests: (1) Wechsler Intelligence Scale for Children for the determination of the intelligence quotient; (2) Tower of London test for the assessment of planning and problem solving skills and cognitive flexibility; (3) span of forward and backward memory numbers (DSF and DSB) for the measurement of short-term verbal memory and working memory; (4) Trail-Making Test Part A and Part B for the evaluation of visual search strategies, selective and divided attention; (5) phonological (FAS) and semantic (CAT) verbal fluency test for the evaluation of verbal ability to access vocabulary by phonological and semantic means; (6) Subtest ToM and ER of the NEPSY-II battery to assess the ability to recognize one's own and others' mental states and the ability to recognize facial expressions; (7) Physical and Neurological Assessment of Subtle Signs to evaluate minor neurological signs; (8) Schedule for Affective Disorders and Schizophrenia for School-Aged Children—Present and Lifetime Version, a psychodiagnostic tool for the assessment of psychopathological symptoms in children and adolescents according to *DSM-IV Text Revision* criteria; (9) Conners' Parents and Teachers Rating Scale–Revised, questionnaires to be filled in by parents and/or teachers, used for the evaluation of ADHD from 3 to 17 years, and for externalizing disorders that can be found in comorbidities; they also provide an index (ADHD Index), which is able to differentiate subjects affected by unaffected; (10) Child Behavior Checklist for Ages 6–18, questionnaire to be filled in by parents, which assesses the presence of internalizing and externalizing symptoms in children and adolescents aged between 6 and 18 years.

### Statistical Analysis

#### Neurophysiological Results

Mismatch negativity and P300 latencies and amplitudes were compared by means of one-way analysis of variance (ANOVA), by considering the group of subjects (iADHD, tscADHD, and HC) as the variable. Significant values underwent *post hoc* Bonferroni test. Moreover, because of the low number of tscADHD patients, as compared to iADHD patients and healthy subjects, we performed also a single-case analysis by considering patients falling within or out of a 95% confidence level interval of any ERP parameter, calculated from healthy subjects.

#### Neuropsychological Results

The scores obtained at the different tests were compared between iADHD and tscADHD patients by means of unpaired Student *t*-test.

#### Correlation Analysis

In our patients, we investigated whether there was a correlation between the ERP data and the neuropsychological results. A series of correlation analyses between the latencies and amplitudes of both the MMN and the P300 potentials and the different neuropsychological scores was performed. Pearson coefficients were computed.

The statistical significance was fixed at *P* < 0.05.

## Results

### Demographics

We enrolled 6 patients (3 females, 6 males) with ADHD secondary to TSC, aged 8–15 years (mean = 12.6 years, median = 15 years), and 13 patients (4 females, 9 males) with idiopathic ADHD, aged 7 to 16 years (mean = 10.4 years, median = 9 years). We also included in the study 14 HCs (9 females, 5 males) aged 8 to 16 years (mean = 11.9 years, median = 12.5 years). No difference in age was found between the groups of subjects (one-way ANOVA: *F* = 0.57, *P* = 0.57).

### Clinical, Neurophysiological, and Neuroimaging Data

In patients with tscADHD, we also collected additional clinical information. None of them presented with active clinical seizures at the moment of the study, and only one of them was under antiepileptic treatment (carbamazepine), with the last epileptic seizure ~2 years before the study enrolment. All but one presented a normal EEG; the only patient with abnormal EEG presented sporadic epileptiform bilateral temporo-occipital abnormalities. All six tscADHD patients presented typical brain magnetic resonance imaging patterns with cortico/subcortical tubers, in all brain areas, including frontal and temporal lobes. None of them presented large, dysplastic, or cystic lesions. None of the patients presented brainstem lesions. They all presented white matter migration lines and subependymal nodules. Subependymal giant cell tumor was present in one patient. Table 1 summarizes main clinical, EEG, and neuroimaging data of tscADHD patients.

**Table 1:** Clinical, EEG, and neuroimaging characteristics of tscADHD patients enrolled in the study.

**Patient**	**Active epilepsy**	**AED treatment**	**IED**	**Brain MRI**			
				**Tubers**	**RML**	**SEN**	**SEGA**
1	No	No	No	Yes, diffuse	Yes, diffuse	Yes	No
2	No	No	No	Yes, diffuse	Yes, diffuse	Yes	No
3	No	No	No	Yes, diffuse	Yes, diffuse	Yes	No
4	No	No	Yes, bilateral TO	Yes, diffuse	Yes, diffuse	Yes	No
5	No	No	No	Yes, diffuse	Yes, diffuse	Yes	No
6	No	CBZ	No	Yes, diffuse	Yes, diffuse	Yes	Yes

*Active epilepsy means epileptic seizures in the last 2 years. AED, antiepileptic drugs; IED, interictal epileptiform discharges; RML, radial migration lines; SEN, subependymal nodules; SEGA, subependymal giant cell astrocytomas; MRI, magnetic resonance imaging; CBZ, carbamazepine; TO, temporo-occipital.

### ERP Results

Event-related potential values are shown in Table 2. N1 (*F* = 1.1, *P* = 0.34) and P2 (*F* = 1.22, *P* = 0.31) latencies and N1/P2 amplitude (*F* = 0.13, *P* = 0.88) were not different between groups.

**Table 2:** Event-related potential amplitudes and latencies in all subjects enrolled in the study.

	**N1 latency (ms)**	**P2 latency (ms)**	**N1/P2 amplitude (μV)**	**MMN latency (ms)**	**MMN amplitude (μV)**	**P300 latency (ms)**	**P300 amplitude (μV)**
**iADHD patients**							
1	109	141	16.9	112.6	10.5	299.8	9.2
2	112.1	165.5	8.7	120.9	4	301.5	5.6
3	108	141.3	11.3	141.1	6.7	330.8	2.2
4	90.8	151.4	10.9	103.5	3.3	319.8	3.7
5	109.1	167.2	13.5	109.1	20.3	327.4	6.5
6	97.9	127	16.9	97.9	7.5	319.1	13
7	109	141.4	13.6	125	33.9	349.1	3.5
8	109.6	159.2	22.6	109.6	12.8	363.3	2.7
9	106.7	139.4	7.8	106.7	6.8	279.3	9.3
10	109	141.3	15.8	148.9	15.4	298.1	9.78
11	117.2	136	13.4	127.9	20.9	325.4	11.5
12	110.4	152.6	14.3	114.5	13	295.9	12.3
13	95	123	10.2	95	5.3	312	5.5
Mean	106	145.1	13.5	116.4	12.3	317	7.3
Standard deviation	7.4	13.5	4	16	8.7	23	3.8
**tscADHD patients**							
1	110.4	145.8	9.2	91.6	4.8	340.1	5.2
2	109	140.3	13.9	105.8	18.2	383.1	26.3
3	108.9	158.7	15.7	143.3	15	264.2	20.2
4	74.7	94.7	18	74.7	11	249.5	18.7
5	115	135.7	14	115	45.4	312.7	7.5
6	104	166.8	12.7	104	14.8	324.2	7
Mean	103.7	140.3	13.9	105.7	18.2	312.3	14.2
Standard deviation	14.6	25.2	3	23.1	14.1	49.4	8.7
**Healthy subjects**							
1	79.9	132	7.6	99.3	5.8	360.3	30.9
2	119.1	148.2	17.1	99	9.8	415	31.3
3	108	174.5	17.1	160	5	317	22.3
4	101	145	11.4	154.7	5	373.8	30.4
5	102	156.5	9.5	168	3.8	276	15.3
6	101.2	122.2	19.3	152	4.5	235	8.9
7	86	180.8	12.2	132.2	3	386	23.4
8	98.8	148	13.3	140	4.4	301.7	7.5
9	124	172.4	17.4	136	5.8	388	9
10	95	132.6	8	60	4.8	401	20
11	95	165.5	16.8	132	1.9	378.5	14.7
12	109	148.6	8	167	10.1	320	13
13	101.1	127	14.7	161.8	6.8	552	17.8
14	95	141.6	13.2	158	3.9	353.2	27.8
Mean	101.1	149.6	13.3	137.1	5.3	361.3	19.5
Standard deviation	11.6	18.3	4	31.4	2.3	75.0	8.5

*Means and standard deviations in each group of subjects are also shown*.

Analysis of variance showed a significant effect of the group on both MMN latency (*F* = 7.5, *P* = 0.01) and amplitude (*F* = 3.2, *P* = 0.0001). *Post hoc* analysis showed that the MMN latency was shorter in patients than in HC, and this difference was significant for iADHD patients (*P* = 0.04) and marginally significant for tscADHD patients (*P* = 0.06). No difference was found between patients' groups (*P* = 0.3). As for the MMN amplitude, it was significantly higher in patients than in HCs (*P* < 0.01). No difference was found between patients' groups (*P* = 0.32).

One-way ANOVA showed a significant effect of the group on the P300 amplitude (*F* = 13.7, *P* < 0.001). *Post hoc* analysis showed the P300 amplitude was significantly lower in iADHD patients than in both tscADHD patients (*P* = 0.03) and HCs (*P* < 0.001). No difference was found between tscADHD patients and HCs (*P* = 0.2). As for the P300 latency, the ANOVA showed a global effect of the group (*F* = 4.3, *P* = 0.02), but the *post hoc* analysis did not show any significant difference.

Table 3 shows a single case analysis for any considered ERP value. This confirms what was found with the ANOVA.

**Table 3:** The 95% confidence level intervals for the different ERP parameters.

	95% confidence level interval^*^	**iADHD**		**tscADHD**	
		**In**	**Out**	**In**	**Out**
N1 latency	91.4–110.7 ms	10	3	4	2
P2 latency	135–164.6 ms	9	4	4	2
**MMN latency**	**115.8–158.4 ms**	**5**	**8**	**1**	**5**
P300 latency	310.5–411.8 ms	8	5	4	2
N1/P2 amplitude	10–16.5 μV	8	5	4	2
**MMN amplitude**	**3.8–6.9** **μV**	**4**	**9**	**1**	**5**
**P300 amplitude**	**13.7–25.2** **μV**	**0**	**13**	**2**	**4**

*The numbers of both iADHD and tscADHD patients falling within (IN) or out of (Out) each interval are indicated*.

* *Calculated from values recorded in healthy subjects*.

*Bold characters indicate the significant differences found with the analysis of variance (ANOVA)*.

**Figure 1:**
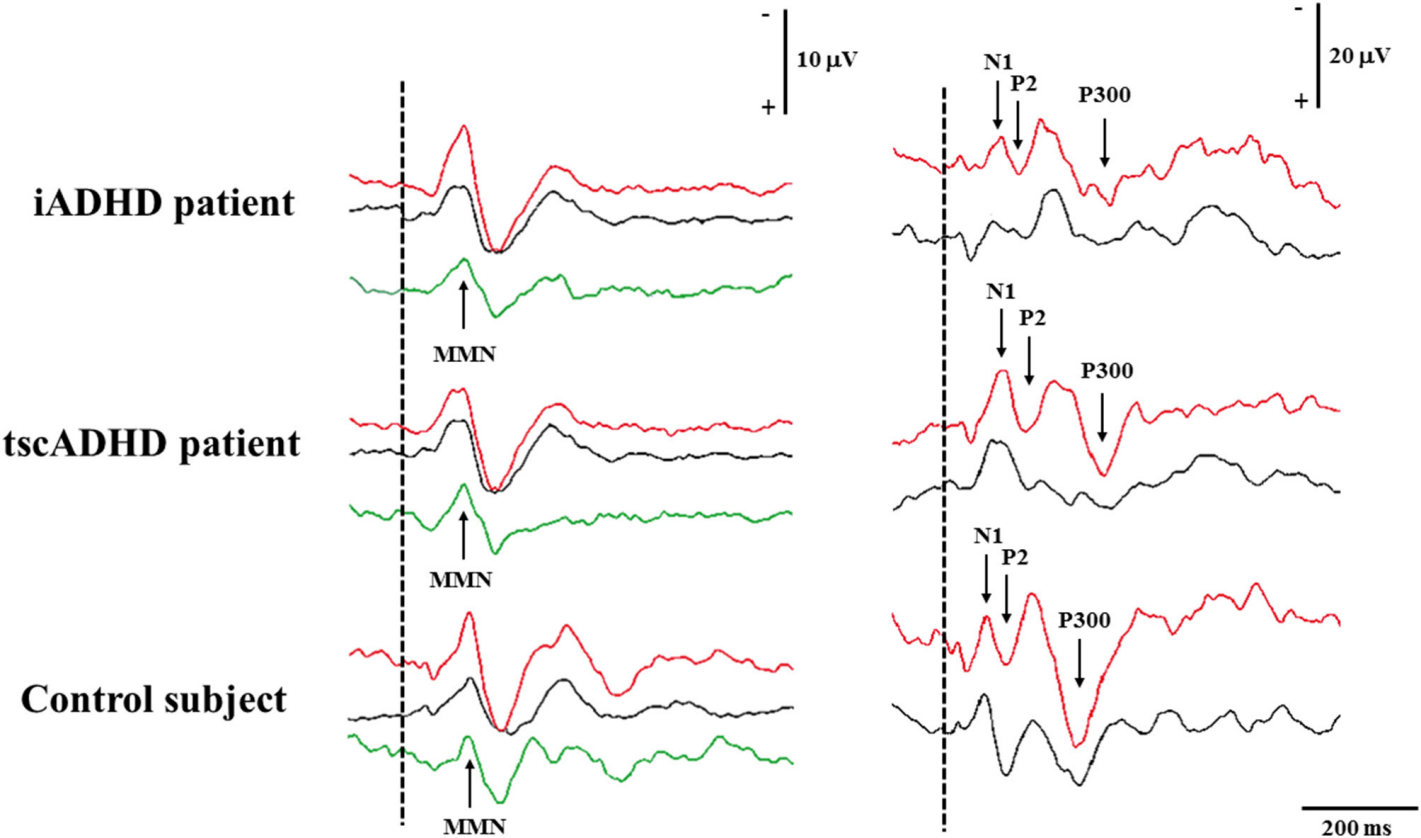
The figure shows MMN (left) and P300 (right) recording in a patient with iADHD (upper), tscADHD (middle), and a control subject (lower). For MMN recording, the Fz traces are shown, whereas for P300 recording the traces are obtained from the Pz electrode. Recordings to frequent and deviant stimuli are in black and red color, respectively, whereas for MMN recording, the green curves are calculated by subtracting traces to frequent stimuli from those to deviant stimuli.

Figure 1 shows P300 and MMN of one patient with iADHD compared with a patient with tscADHD and a control subject.

### Cognitive and Neuropsychological Results

#### Cognition

The average intellectual quotient (IQ) values were overall lower in tscADHD than in iADHD patients, with statistically significant differences in all the quotients, so total IQ (105.9 ± 10.2 vs. 85.8 ± 16.4; *P* = 0.004), verbal IQ (113.9 ± 15.2 vs. 92.2 ± 15.9; *P* = 0.016), and performance IQ (109.1 ± 15.6 vs. 91.3 ± 12.2; *P* = 0.032) (Table 4).

**Table 4:** Mean values of total (TIQ), verbal (VIQ), and performance (PIQ) intelligent quotient in patients with idiopathic ADHD compared to patients to ADHD associated with TSC.

	**iADHD**	**tscADHD**	***P***
TIQ	105.9	85.8	0.004
VIQ	113.9	92.2	0.016
PIQ	109.1	91.3	0.032

#### Neuropsychological Data

Performances in executive functions were globally lower in subjects with tscADHD than in iADHD patients, with statistically significant differences both in direct number span (DSF) (*P* = 0.006) and in categorical fluency (CAT) (*P* = 0.018). Although tscADHD children performed worse in all the other administered tests, no other statistical differences have been detected. Table 5 summarizes the results of neuropsychological assessments.

**Table 5:** Mean values of the results obtained in neuropsychological tests administered in subjects with idiopathic ADHD and ADHD with TSC.

	**iADHD**	**tscADHD**	***P***
ToL (z)	−0.7	−1.22	0.08
DSF	−0.43	−2.15	**0.006**
DSB	−0.05	−1.22	0.05
FAS	−1.2	−1.24	0.87
CAT	−1.7	−3.51	**0.018**
TMTA (s)	62.4	81.33	0.47
TMTB (s)	148.3	114	0.32
CPRS- opp	60.76	66.83	0.43
CPRS-inatt	74.61	80.33	0.5
CPRS-hyper	68.53	70.16	0.83
CPRS-ADHD index	76.61	78.66	0.75

*Significant P values in bold. ToL, Tower of London; DSF, direct number span; DSB, span of inverse numbers; FAS, phonemic fluence; CAT, categorical fluency; CAT TMTA, Trail-Making Test Part A; TMTB, Trail-Making Test Part B*.

#### Correlation Analysis

**Figure 2:**
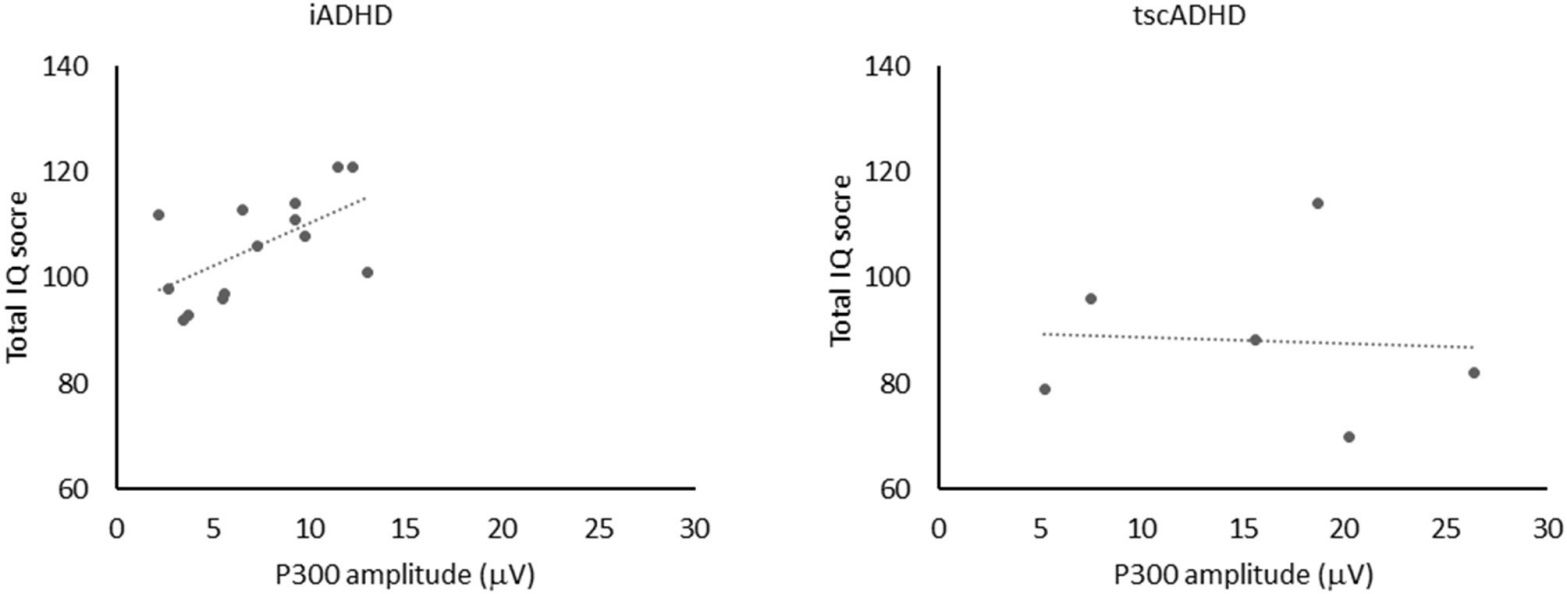
The figure shows the positive correlation (left) between P300 amplitude and total IQ score in iADHD patients (*R*^2^ = 0.35, *P* = 0.03). No significant correlation (right) between P300 amplitude and total IQ score was found in tcsADHD patients (*R*^2^ = 0.004, *P* = 0.9).

We found one significant positive correlation between the P300 amplitude and total IQ score in iADHD patients (*R*^2^ = 0.35, *P* = 0.03). It means that in this group of patients the larger was the P300 amplitude, the higher was the total IQ (Figure 2). It is noteworthy that the same correlation was not found in tscADHD patients (R^2^= 0.004, *P* = 0.9). No other significant correlations were obtained.

## Discussion

To the best of our knowledge, this is the first study comparing neurophysiological and neuropsychological tests between patients with iADHD and tscADHD. The main difference between the groups of patients concerned the P300 amplitude, which was lower in iADHD but not in tscADHD patients, as compared to HC. This difference allows us to speculate about the psychophysiological meaning of the P300 amplitude in different clinical contexts.

### P300 Amplitude in ADHD May Depend on the Etiology

That patients with idiopathic ADHD present a significantly lower P300 amplitude than HCs, which appears to be in good agreement with previous reports (22), suggesting reduced attentional orienting to warning stimuli in patients with ADHD when compared to normal subjects. Indeed, reduced P300 amplitude has been interpreted as a failure to allocate sufficient attentional resources to stimulus evaluation processes due to reduced attentional capacity (23). On the other hand, although the mean P300 amplitude of tscADHD patients was slightly lower than that observed in healthy children, this difference did not reach the statistical significance. These results might be difficult to be interpreted, because we could expect that tscADHD patients, expressing more severe ADHD symptoms, could present more significant abnormalities. However, it is important to note that, although most of literature data suggest that children with ADHD show lower P300 amplitudes, there are some conflicting results failing to replicate these findings (24). Furthermore, in our sample of iADHD children, the P300 amplitude showed a linear correlation with the cognitive level, which was not found in subjects with TSC. Our results therefore highlight that, although ERPs are a very interesting technique to study brain functioning in neuropsychological dysfunctions, P300 amplitude might be influenced by different variables, especially by cognitive factors (20), which prevent it to be used as a reliable marker of attention at least in some neurological conditions. Although this cannot be proven, we hypothesize that the marked network dysregulation typical of children with TSC might be responsible of this lack of association and that in such a brain disease P300 amplitude might not be a real marker of attention processes. In addition, in the interpretation of these data, we cannot exclude that results might have been influenced by patients' age. Indeed, a longitudinal study showed that after the first decade of life up to young adulthood, the P300 difference between ADHD and non-ADHD groups is no longer significant (25). Seen in this light, we must underline that in our sample tscADHD patients presented a higher mean age, with only two patients younger than 10 years, as compared to iADHD patients, who included 7 of 13 subjects younger than 10 years.

### MMN in ADHD

As for MMN, patients with ADHD (both with and without TSC) presented statistically significantly higher amplitudes and lower latencies, without differences between children with and without TSC. It is to be underlined that the shorter MMN latency in ADHD patients could not be attributed to a peripheral event, because both the N1 and P2 latencies were not different between our groups (iADHD and tscADHD patients and healthy subjects). Although most of literature data suggest that children with ADHD present a lower MMN amplitude and an increased MMN latency (9, 18), there are also studies revealing no significant differences (19)or an increased MMN amplitude in ADHD (26). Furthermore, similarly to what has been reported for P300, also the differences in MMN amplitude between children with ADHD and HCs tend to decrease with age (27). Whatever the reason of the disagreement between the present results and those of previous studies, it is conceivable that in patients with ADHD, who hardly keep their attention focused on one target and are easily distracted by unexpected stimuli, the MMN component shows shorter latency and higher amplitude than in HC. Mismatch negativity component is thought to reflect the preattentive detection of deviants (28). Despite MMN is mainly generated within the bilateral temporal cortex, it was shown that a further frontal source is activated when the preattentive processing of deviants is made difficult by a concomitant highly demanding task (29). This frontal contribution to the MMN building can result in a higher late MMN amplitude (29). Seen in this light, the hypothesis can be made that in ADHD patients, whose attentional resources are lower than in HC, even a simple task (reading a novel), as that used in the present study, can lead to the same result as a task requiring a far larger attentional load.

Although P300 amplitude differentiated iADHD from tscADHD patients, it is to be underlined that the MMN behavior was similar in both groups of patients. Indeed, both iADHD and tscADHD patients showed an MMN component shorter in latency and higher in amplitude as compared to HC. This finding can be relevant for two main reasons. First, from a clinical point of view, it suggests that the MMN characteristics are more dependent on the brain dynamics typical of ADHD rather than on ADHD etiology. This means that in the neurophysiological assessment and follow-up of ADHD patients MMN can provide particularly useful information. Second, the pathophysiological mechanisms of ADHD, which are far to be completely understood (30), involve the brain circuits subtending the involuntary attention, whose neurophysiological marker is represented by MMN.

### Limitations of the Study

This study has some limitations that must be considered. First, we are aware that the sample studied is limited, but TSC is a rare disease, with a very high range of neuropsychiatric comorbidities; therefore, finding a homogenous sample of patients in a specific age range has been quite difficult. Furthermore, all patients with tscADHD presented structural brain lesions in both the gray and white matter, and one tscADHD patient was assuming an antiepileptic drug. We cannot exclude that these elements can have influence the ERP pattern in our tscAHDH patients. As for the typical TSC brain lesions, a control population of subjects affected by TSC but without presenting ADHD symptoms could have helped us in disentangling the contribution of ADHD and that of TSC to the observed findings. Future studies will be hopefully addressed to clarify this issue. As for the single tscADHD patient under antiepileptic treatment, it is to be underlined that his exclusion from the statistical analysis did not change our results.

### Conclusions

While P300 amplitude is commonly considered a neurophysiological marker of the efficiency of the attentional processes, our data suggest that in specific clinical contexts, such as TSC patients, the impairment of the cognitive functions might not be reflected by reduced P300 amplitude. This can occur when the cortical networks underlying the attentional mechanisms are particularly disrupted, as in TSC.

## Data Availability Statement

The original contributions presented in the study are included in the article/supplementary materials, further inquiries can be directed to the corresponding author/s.

## Ethics Statement

The studies involving human participants were reviewed and approved by EB of bambino gesù children's hospital. Written informed consent to participate in this study was provided by the participants' legal guardian/next of kin.

## Author Contributions

RM and MV: first conceptualized the work. RM and SM: enrolled the patients. SM: performed neuropsychological examinations. SM, DD, and SP: performed neurophysiological examinations. RM: wrote the first draft. PC, SP, MV, and FV: performed the corrections on the different versions of the draft, revised the literature, and updated the manuscript. All the authors approved the final version of the manuscript.

## Conflict of Interest

The authors declare that the research was conducted in the absence of any commercial or financial relationships that could be construed as a potential conflict of interest.
